# Announcement: International Symposium on Metals and Genetics

**DOI:** 10.1155/MBD.1994.337

**Published:** 1994


					INTERNATIONAL SYMPOSIUM
METALS AND GENETICS
Toronto, May 24-27,1994
ON
Location and Dates:
The symposium will be held in Toronto, Canada, from 24- 27 May, 1994. As one
of the academic and cultural centers of Canada, Toronto boasts restaurants,
theaters, museums, art galleries, and sights that can be counted amongst the
world's finest.
Aims:
It is the aim of this conference to bring together investigators from diverse fields in
order that the broad implications of the direct and indirect interactions of metals
and DNA may be discussed. The conference will be organized around
approximately six areas involving metals and genetics:
I. Molecular mechanisms of metal induced mutagenicity and
carcinogenicity: It has been known for several years that metals such as nickel
and chromium can cause cancer. The latest advances which have begun to
demonstrate the underlying mechanisms will be discussed.
II. Metal induced DNA cleavage: Recent investigations have shown that
synthesised metal compounds, hybrid proteins and existing metal binding
proteins are able to cleave DNA. At this conference, the design of such agents,
their mechanisms of action and possible applications will be discussed.
III. Genetics and Biochemistry of Metal Related Diseases: The
identification of the genes for Menkes and Wilson diseases will be discussed.
Advances in the understanding of the pathophysiology of these diseases as well
as advances in therapies for these diseases will be examined.
IV. Zinc Finger Proteins: The structures and functions of this unique class of
DNA bindmg proteins will be examined.
V. Metal Effects on Gene Expression: In this conference the mechanisms of
metal induced gene expression will be discussed.
VI, Direct Interaction of Metals and DNA" In this area the direct binding of
metals to DNA, including the mechanisms and the structural aspects of this
binding will be examined.
Further details and/or enquiries should be
forwarded to:
Dr. B. Sarkar, Chairman
International Symposium on Metals and Genetics
Head, Department of Biochemistry Research
The Hospital for Sick Children
555 University Avenue
Toronto, Ontario, Canada M5G 1X8
Fax: (416) 813 5379
337

				

